# Effects of Tensile Specimen Geometry and Gripping System on the Mechanical Stability of Ausferrite in Austempered Ductile Irons

**DOI:** 10.3390/ma18184359

**Published:** 2025-09-18

**Authors:** Lun Fu, Manuel Schiralli, Maurizio Vedani, Jakob Olofsson, Marcin Górny, Parnian Govahi, Riccardo Donnini, Maria Losurdo, Giuliano Angella

**Affiliations:** 1 College of Mechanical Engineering, Taiyuan University of Technology, Taiyuan 030024, China; fulun@tyut.edu.cn; 2 National Key Laboratory of Metal Forming Technology and Heavy Equipment, Taiyuan University of Technology, Taiyuan 030024, China; 3 Department of Mechanical Engineering, Politecnico di Milano, Via La Masa 1, 20156 Milan, Italy; manuel.schiralli@mail.polimi.it (M.S.); maurizio.vedani@polimi.it (M.V.); 4 Department of Materials and Manufacturing, School of Engineering, Jönköping University, Box 1026, SE-551 11 Jönköping, Sweden; jakob.olofsson@ju.se; 5 Faculty of Foundry Engineering, AGH University of Krakow, ul. Reymonta 23, 30-059 Krakow, Poland; mgorny@agh.edu.pl; 6 Research Institute CNR-ICMATE, Unit of Milano, via R. Cozzi 53, 20125 Milan, Italy; parniangovahi@cnr.it (P.G.); riccardo.donnini@cnr.it (R.D.); maria.losurdo@cnr.it (M.L.)

**Keywords:** austempered ductile iron, ausferrite instability, tensile specimen geometry, tensile testing

## Abstract

Different combinations of round and flat tensile specimens for different gripping systems of Austempered Ductile Irons (ADIs) were produced from the same 25 mm Y-block castings to investigate the effect of the specimen geometry and gripping system on the tensile mechanical properties of ADIs. Particular attention was paid to the analysis of strain-hardening behavior of ADIs that can be related to the stability of ausferrite, when austenite transforms into martensite. Moreover, Digital Image Correlation (DIC) was carried out on the flat tensile specimens to analyze the strain distribution of the material in real time. To quantify the austenite stability with plastic deformation, X-ray Diffraction (XRD) analysis was performed on ADIs before and after straining. Finally, Finite Element Modeling (FEM) simulations were carried out to analyze the stress distribution along the tensile specimens in all the different tensile testing configurations (tensile specimen geometry + gripping system). The flat specimens showed lower ductility and higher strain-hardening rates; however, the flat tensile specimens with the wedge gripping system experienced the highest strain-hardening rate, suggesting a significant decrease in the ausferrite stability in this tensile testing configuration. FEM simulations showed that the specimen geometry and the gripping system influenced the tensile behavior of ADI by reducing the ductility because of stress intensification and triaxiality effects. Furthermore, the stress intensification and triaxiality factor caused a higher strain-hardening rate, which was associated with increased ausferrite instability.

## 1. Introduction

Austempered Ductile Irons (ADIs) are advanced nodular cast irons produced through heat treating conventional DIs. Their exceptional mechanical properties result from the formation of a dual-phase acicular microstructure known as ausferrite, composed of hard bainitic ferrite (α) and metastable austenite with a high carbon content (γ_HC_). The final microstructure of ADI consists of plate-like ferrite, carbon-enriched retained austenite, and nodular graphite [1,2,3,4,5,6,7,8]. This unique microstructure provides ADI with superior mechanical properties compared to conventional DIs, including enhanced yield strength, good ductility, increased wear resistance [9,10,11,12,13,14,15], as well as good impact properties [16,17,18,19,20], machinability [21,22], improved fatigue resistance [23,24] and fracture toughness [14,15,25,26,27]. Furthermore, as its density is 10–12% lower than steels with similar mechanical properties [7,14,15], ADIs are competitive as potential substitution for production of industrial components [28,29]: the lower hardness grades (ADI JS 800-10, and 1050-6) have good fatigue properties, so they can be used in shafts, hubs, suspension arms, steering knuckle, brake calipers in automotive; wheel centers, bogie frames and couplers for heavy haul operations in railway; armor applications, particularly against small arms fire, perforated plates and ground-engaging tools in military vehicles for defense applications. The higher hardness grades (ADI JS 1400-1, HBW450 and ADIWRPAT) are abrasive wear-resistant, so they can be used in mining and other wear applications, like bulldozers and excavators.

The ADI production process involves a two-stage heat treatment: a conventional DI is first austenitized in the temperature range of 850–890 °C; then, it is isothermally held in a salt bath at temperatures typically ranging from 250 to 400 °C to enable the austempering transformation γ → α + γ_HC_. During this stage, known as ausferritization, ferrite laths emerge from the grain boundaries of the metastable austenite. Due to its lower carbon solubility compared to austenite, ferrite releases excess carbon that then diffuses into the surrounding austenite. As more carbon diffuses into the austenite, its stability increases, allowing it to persist even at room temperature. After ausferritization, the system is slowly cooled to room temperature to prevent the formation of residual stress. The austempering time window is critical: if the ausferritization is stopped earlier than the optimal time, unstable austenite is produced that can transform easily into martensite under thermal and mechanical loading. On the other hand, prolonged exposure to the austempering temperature can cause the decomposition of austenite into ferrite and FeC carbide (γ_HC_ → α + ε’), leading to a significant decrease in the ductility of ausferrite. Therefore, to produce ausferrite with an optimal balance of mechanical properties, it is essential to find a suitable austempering time window that maximizes the austempering reaction (γ → α + γ_HC_) while avoiding the detrimental reaction (γ_HC_ → α + ε’) [1,2,3,4,5,6].

There are several factors that can affect the austenite-to-martensite transformation, such as chemical composition, heat treatment parameters, temperature and mechanical loading conditions, to name a few [6,30,31,32,33]. In [34], the effect of heat treatment parameters on the austenite’s ability to undergo strain-induced martensite transformation was investigated. The authors showed that the most favorable conditions for austenite-to-martensite transformation in ADIs happen after ausferritization at high temperatures, from 370 °C to 410 °C for 60 min to 120 min, after austenitization at temperatures lower than 900 °C. Also, austenitization temperature is relevant to the stability of austenite, which is often underestimated [33]. Moreover, lower carbon content in austenite reduces the strain required to mechanically induce the martensite formation, thereby lowering the stability of the ausferrite. If the carbon level is low enough, austenite can transform into martensite under stress even before plastic deformation begins, significantly decreasing ductility. In fact, as the free energy of Gibbs of austenite is higher than that of martensite at room temperature, the retained austenite is metastable. To trigger the austenite-to-martensite transformation, the temperature should be lowered to a temperature where the difference in free energy between austenite and martensite is so high to drive the diffusionless transformation: this temperature is the martensite-start-temperature Ms [35,36]. For temperatures slightly higher than Ms, the addition of little mechanical energy with tensile (or compression) loading triggers the austenite-to-martensite transformation at a critical stress σ_C_ lower than yielding, then austenite to martensite is stress-induced, i.e., activated only by elastic mechanical energy; otherwise, with increasing temperatures, the transformation occurs for σ_C_ higher than yielding and is called strain-induced. The effect of tensile test temperature on the formation of strain-induced and stress-induced martensite was investigated in [8,14,15,37,38], and in [39], where an ADI1100 was tensile tested in a wide range of temperatures between −70 °C and 200 °C, experiencing both conditions. Stress-induced martensite developed at lower temperatures or under high triaxiality factors, such as those experienced in standardized specimens for fracture toughness tests [27], leading to low ductility, while strain-induced martensite formed at higher temperatures.

Tensile testing can be a useful tool to assess the ausferrite stability in ADIs [12,13,40,41,42]. Angella et al. [43] observed that specimens’ geometry seems to influence austenite stability, with flat specimens showing more ausferrite instability compared to round specimens; however, the investigations were limited to two tensile specimen geometries, with no considerations given to the gripping systems, which are a significant component of tensile testing. The present work studies the effect of tensile specimen geometry and tensile gripping system on the tensile mechanical properties of ADIs. Round and flat tensile specimen geometries were used in combination with thread, pin-hole and wedge grips. A Digital Image Correlation (DIC) system was used to collect local information on the stress–strain conditions during tensile testing of flat geometries. Moreover, XRD measurements were carried out to follow the austenite-to-martensite transformation during straining.

## 2. Materials and Methods

### 2.1. ADI Production

ADIs with a minimum UTS of 800 MPa (ADI800) and two different chemical compositions (ADI_1 and ADI_2, reported in Table 1) were tested in tension. The chemical compositions of the two investigated ADIs differed mainly in the content of Ni and are reported in Table 1. The use of two different compositions was only due to the limited amount of material available from a single heat. To produce tensile specimens with different geometries from the same heat, additional material was needed. Therefore, two heats were used, not for comparing compositions, but only to ensure an adequate number of specimens. This allowed the study to focus on the effect of specimen geometry and/or gripping systems, while keeping the material composition as consistent as possible. Finally, tensile specimens with different geometries made of high silicon ductile iron (4.2%wt. Si, HSi reported in Table 1) were also tested to evaluate the effects of a ferritic metallic microstructure, which is stable at room temperature. Details about the production and characterization of HSi 4.2%wt. of Si are reported elsewhere [44]. The original DIs for subsequent austempering were produced into a casting wall section of 25 mm complying with Y-blocks ASTM A536–84(2019)e1 [45]. Round and flat tensile specimens were machined from the same heat.

**Figure 1:**
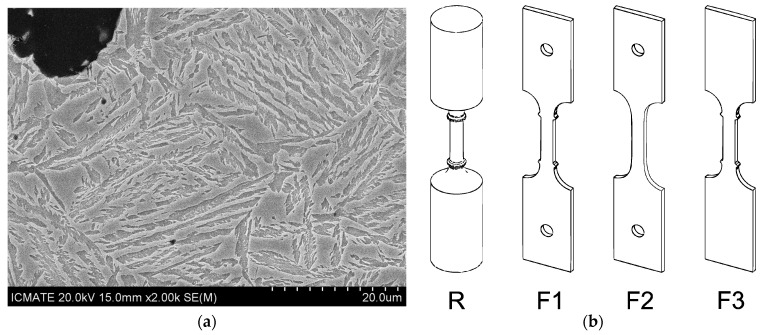
(**a**) Ausferritic matrix in ADI800 with chemical composition ADI_2; (**b**) the different geometries of tensile specimens investigated, all complying with ASTM E8/E8M-11 [47].

Experimental melts were carried out in medium-frequency induction furnaces with a capacity of 12 tons. The charge consisted of 60% circulating own scrap and 40% steel scrap, with carburizer to correct the content of C. At 1420 °C, after the slag was removed from the melt surface, the melt was poured into green sand molds. The inoculation was carried out in the stream, and the spheroidization in the ladle. The castings were then austenitized at 875 °C for 2 h, and austempered at 380 °C for 1 h, resulting in graphitic nodules embedded into ausferritic matrix with metastable austenite rich in C (*γ*_HC_) and bainitic acicular ferrite (*α*). ADI_1 and ADI_2 had a similar graphitic structure as they were produced with the same Y-block geometry of 25 mm, with a nodule count of about 300 n/mm^2^, and nodularity higher than 90%, so, well above the threshold reported in ASTM A247-19 for good nodularity, i.e., 80%. Details of the graphitic structure and metallic matrix are reported in [43]. Figure 1a shows an example of ausferritic microstructure of the ADI_2 composition, containing 1.5%wt. of Ni, after chemical etching with 5% Nital (nitric acid in ethanol). The etched acicular phase is bainitic ferrite (*α*), while the coarse gray phase is the austenite rich in carbon (*γ*_HC_). HSi ductile iron with the nominal content of 4.2%wt. of Si was produced into Y-blocks with wall thicknesses of 75 mm, complying with ASTM A 536-84(2019)e1 [45]. The melt was poured into a green sand mold at 1420 °C with stream inoculation and ladle modularization, and no chemical adjustment was made to modify the graphitization potential. The assessment of the carbon-equivalent value C_Eq_ depends on the chemical elements of the melt, mainly C, Si and P, according to linear equations. Indeed, the equation coefficients are not constant and depend on the element ranges, so for the chemical composition of ADIs with low silicon content, C_Eq_ was calculated according to [43], while for higher Si content, C_Eq_ was calculated according to [46], as reported in Table 1.

### 2.2. Tensile Testing

Four series of ADI specimens reported in Figure 1b were prepared for tensile testing, all complying with ASTM E8/E8M-11 [47]: (1) round specimens with ridges and threaded heads, (2) flat specimens with ridges and pin-hole gripping systems, (3) flat specimens without ridges and pin-hole gripping systems and (4) flat specimens with ridges and wedge gripping systems. Additionally, two other series of tensile specimens made of high-silicon ductile iron (HSi 4.2%wt. of Si) were tested with round tensile geometry and threaded heads and flat geometry with ridges and pin-hole gripping systems.

For simplicity, these tensile specimens’ geometries were referred to as ADI-R, ADI-F1, ADI-F2 and ADI-F3, respectively, and as HSi-R and HSi-F3 for the cast iron HSi4.2%wt. round and flat specimens, respectively. In Table 2, detailed descriptions of the geometry and gripping system of each type of tensile specimen are reported. All ADI and HSi tensile specimens were tested in strain control mode with a strain rate of 10^−4^ 1/s complying with ASTM E8/E8M-11 [47]: for the tensile round geometry, the gauge diameter and gauge length were *d*_o_ = 5.6 mm and *l*_o_ = 28 mm, respectively. For the flat specimen, the gauge thickness was *t*_o_ = 1.5 mm, width *w*_o_ = 5.0 mm and length *l*_o_ = 20.0 mm. Furthermore, ADI-F3 and HSi-F3 specimens were tested with a wedge gripping system and were equipped with DIC, while all the other specimens were tested with conventional elongation measuring systems. Specifically, the tensile specimens with ridges were tested with elongation control carried out by using LVDT transducers connected to the stems that were rigidly attached to the ridges by means of collars, while the ADI-F2 specimens without ridges were tested in stroke control. For each combination of tensile specimen geometry and gripping system with different chemical compositions (ADIs and HSi), at least four tensile specimens were pulled.

The engineering tensile data, including stress *S = F/A*_o_ (MPa) and strain *e* = (l − *l*_o_)/*l*_o_, were calculated using the applied load *F*, the initial cross-sectional area *A*_o_, the initial gauge length *l*_o_ and the instantaneous gauge length *l*. These data were converted into true stress *σ* vs. true strain *ε* values using the standard relationships *σ* = *S*∙(1 + *e*) and *ε* = ln(1 + *e*). This approach was adopted to analyze all results from different pulling configurations, enabling their comparison. For the strain-hardening analysis, only the plastic component of strain (*ε_p_*) was used, defined as *ε_p_* = *ε* − *σ*/*E*, where *E* is the experimental Young modulus. As reported in [12,13], the procedure to find the Voce equation parameters is based on the analysis of the differential data *dσ/dε_p_* vs. *σ*, where *dσ/dε_p_* is the strain-hardening rate. According to the Voce constitutive model of flow stress [42,43,44], strain-hardening rate and stress are linearly related:

(1) $$\frac{d\sigma }{d\varepsilon_{p}}=\Theta_{o}-\frac{\sigma }{\varepsilon_{c}}$$

**Figure 2:**
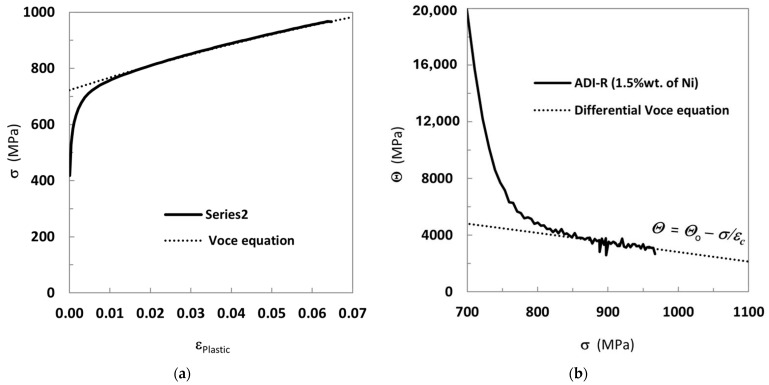
Example of tensile plastic behavior fitting with Voce equation [43]: (**a**) tensile flow curve with round tensile specimen of ADI-R (ADI_2, 1.5%wt. of Ni); (**b**) differential data from the flow curve in a) and strain-hardening analysis based on Voce equation with best linear fit at high stresses.

*Θ*_o_ and 1/*ε_c_* are constants with physical meaning that describe the micro-mechanics of plastic deformation and are related to the matrix microstructure. An example of strain-hardening analysis based on the Voce equation is reported in Figure 2a, where a wide range of experimental data at high stress levels is found.

Through plotting 1/*ε_c_* vs. *Θ*_o_ of a statistically meaningful set of flow curves, the Matrix Assessment Diagram (MAD) is built up, with which the cast iron can be classified, and its quality can be assessed. When Voce tensile data from an ADI produced through different austempering times are plotted versus austempering parameters, an evaluation of the optimal time for austempering reaction can be made [12,13]. For instance, in [12], different austempering times were imposed on the same DI with constant chemical composition and plotted versus the austempering times: the most stable ausferrite produced with the optimal austempering time had the lowest Voce parameters. In [13], DIs with different chemical compositions were heat-treated with the same austenitization route and different austempering conditions, and the most stable ausferrite was the one that, when tensile tested, had the lowest Voce parameters in MAD. So, the stability of ausferrite is related to low Voce parameters, and the strain-hardening analysis via the Voce equation and MAD gives an indication of that. In [43], different ADIs were tensile tested with two different tensile specimen geometries, and gave different Voce parameters, i.e., higher for the flat tensile specimens, suggesting that this tensile geometry could enhance the instability of ausferrite. Unfortunately, the number of tensile specimens was limited; so, in the present investigation, the number of tensile tests was increased. Additionally, the number of possible pulling configurations was increased, considering also different gripping systems, and for each pulling configuration, i.e., tensile specimen geometry + gripping system, four specimens were tested, producing a more statistically meaningful set of data to investigate the stability of ausferrite.

### 2.3. DIC Measurements

To enable DIC measurements, a pattern was manually sprayed on the surface of the specimen, starting with a white bottom layer, followed by a black random speckle pattern. The samples were tested within an hour of color application to avoid decohesion between the metal and the paint. A Stereo-DIC setup was used to observe the deformation of the speckle pattern. The use of a Stereo-DIC setup ensures that out-of-plane motions are also identified in the measurements. Two FLIR cameras with 5 megapixel resolution were used, equipped with Fujinon 25 mm lenses. The cameras were mounted on a bar, with one camera perpendicular to the observed specimen surface and the second at the same height with an angle of about 20°. Stereo calibration was performed before each test. Images were captured at a frame rate of 1 Hz. The MatchID™ Grabber software was used for capturing the images, and the correlation analysis was performed with MatchID™ Stereo. The noise level was measured to be about 0.7%. For displacement calculations, a subset size of 21 pixels and a step size of 10 pixels were used. The spatial resolution for displacements was 21 pixels, or about 0.45 mm. For strain calculations, a quadratic polynomial (Q4) interpolation method was used with a Virtual Strain Gauge Size of 81 pixels (around 1.8 mm) and a logarithmic Euler–Almansi strain tensor.

### 2.4. XRD Measurements

To quantify the volume fraction of austenite before and after straining, X-ray Diffraction (XRD) measurements were carried out on selected flat specimens at three locations: the specimen head, the gauge length and the fracture surface. XRD analysis was carried out using a Siemens D500 diffractometer equipped with Bragg–Brentano geometry and Cu Kα radiation (λ = 0.1542 nm). Data were collected over a 2*θ* range of 20° to 110°, using a step size of 0.02°, and a dwell time of 5 s per step. The volume fractions of austenite and ferrite phases were determined in compliance with ASTM E975-13 [48]. Peak intensities and 2θ positions were identified for the (111)γ, (200)γ, (311)γ, (110)α, (200)α, (211)α and (220)α lattice planes using peak fitting based on the Pearson equation [49]. The effective interaction volume between the X-ray beam and the analyzed material was assessed using the following equation:

*I* = *I*_o_∙exp(−*μ*∙*x*) (2)

where *I*_o_ is the initial beam intensity of X-ray (Cu Kα), *I* is the beam intensity at depth *x* and *μ* is the linear attenuation coefficient, which depends on Cu Kα energy and the material. Considering a value of *μ* equal to 300 cm^−1^ for Cu Kα and Fe [50], and an absorption of 90%, the depth of the surface layer involved in the diffraction measurements was 3.5 μm. The diffractions were carried out only on the surfaces of the tensile flat specimens and for the composition ADI_2 (1.5%wt. of Ni). Since the flat tensile specimens were machined in the same way, any surface microstructure alteration or residual stress due to the machining was the same in all specimens.

The austenite-to-martensite transformation has been widely investigated through X-ray diffraction technique [51,52,53,54,55], as well as through more advanced techniques, like neutron diffraction [56]. Ausferritization is very sensitive to chemical composition, so segregations can be an issue in the formation of stable austenite and uneven distribution of austenite and ferrite/martensite in ausferrite [8]. The flat tensile specimens were machined off through slicing the ADI 25 mm Y-blocks through electron discharge machining, so possible macroscopic microstructure heterogeneities between flat tensile specimens were reduced at least. However, several elements, like Mn, for instance, segregate at the Last Zones to Freeze (LZFs), so the average distance between LSFs can be assumed as the characteristic distance between austenite inhomogeneities. As the LZFs are roughly in between the average internodular distance, the characteristic distance between LZFs is about the average grain size, for 1.5%wt. of Ni (ADI_2) was assessed to be 93.5 μm for the 25 mm Y-block investigated in the present work; see [43]. The surface area that is hit by the XRD beam during diffractometry is about 1 cm^2^, so eventual microstructure uneven distributions because of chemical segregations could not be detected and did not affect the calculation of the ADI austenite volume fractions based on the XRD measurements on the flat specimens’ surface. The relative ratios between the diffraction γ_HC_ and α peaks did not reveal any texture, which is typical of correctly inoculated foundry materials. However, the aim of the diffraction measurements was to identify relative variations in volumetric fractions of retained austenite γ_HC_, which was possible from a single tensile geometry.

### 2.5. FEM Simulations

**Figure 3:**
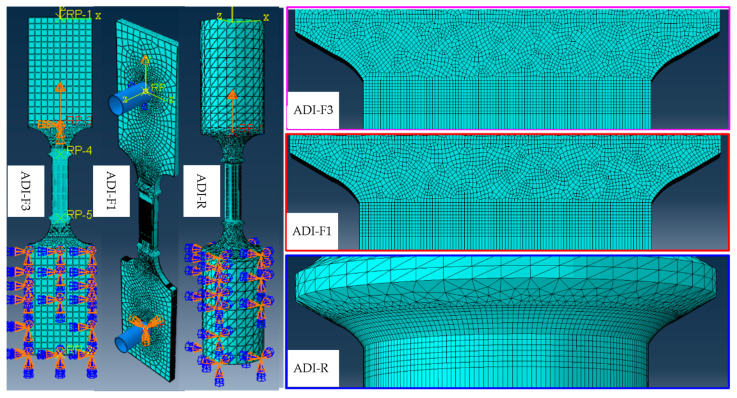
Constraint configuration in FEM simulations for the three different tensile specimens’ geometries and meshing close to the ridges, where stress intensifications were expected.

The 3D FE models, carried out through ABAQUS/CAE^®^, of the three tensile test specimens (ADI-F1, ADI-F3 and ADI-R) for FE simulation analysis are reported in Figure 3. Only the ADI800 with the chemical composition of ADI_2 (1.5%wt. of Ni) was modeled, by using the experimental flow curve in Figure 2a and employing the von Mises yield criterion to describe the plastic yield behavior of the material. So, the constitutive model did not take into account the martensitic transformation. In fact, the core objective of this model was to separate the influence of stress concentration caused by the different tensile specimens’ geometries and the different gripping systems on the fracture behavior from the effects of the austenite instability, thus simplifying the interpretation of the austenite-to-martensite transformation contribution to the tensile plastic behavior process and accompanying the rationalization of the austenite-to-martensite contribution only through experimental means.

Refining the mesh at key locations of tensile specimens was carried out to obtain more accurate results: a sketch of the meshing for the three tensile specimen geometries, close to the ridges where stress intensifications were expected, is reported in Figure 3. When dividing the mesh, local refinement methods were used via 8-node 6-hedral 3-dimensional solid elements (C3D8R) and setting the elements in the cell properties to be deleted. Dynamic display analysis was used, and the analysis step time was 0.04 s, the maximum time increment step 10^−7^, and the mass scaling factor 100. The boundary conditions applied to the models were one fixed tensile specimen end with the displacement load applied to the other end. Specifically, both in ADI-F3 and ADI-R, the nodes of the grips at the top end were coupled to the reference point RP-1, and then a displacement was applied to the reference point. The bottom part was set with completely fixed boundary conditions. In ADI-F1, a rigid pin was added at the hole position, and the rigid pins on the upper and lower sides were all coupled to a reference point. A displacement constraint was applied to the upper reference point, and a completely fixed constraint was applied to the lower reference point, as reported in Figure 3. In the grip section, a coarser mesh size was used to reduce the calculation time. The calculations were carried out through both the dynamic/explicit models. Finally, the dynamic process of the tensile test was carried out, and the stress–strain analysis results were obtained.

The simulation process came into different stages: mainly pre-processing, analysis, and calculation, and, finally, result analysis. First, a pre-processing step was carried out to model the three different tensile specimen geometries. Then, the material properties of ADI800 with the chemical composition of ADI_2 (1.5%wt. of Ni) were assigned, including the Young modulus (180 GPa), Poisson’s ratio for the elastic stage (0.27) and density (7.5 × 10^9^ g/mm^3^). The material fracture parameters were inputs to the simulations. As the influence of different specimen geometries on the final elongation had to be analyzed, the plastic flow curve from the round tensile specimen with the best elongation was taken as the basic material parameter for the simulations of all the different tensile specimen configurations. So, the stress–strain data came from the experimental test on ADI_2 round tensile specimen (ADI-R), i.e., the true stress–strain curve in Figure 2a, where 0.064 was the final plastic strain that was assumed as the fracture plastic strain. The residual fracture energy referred to the energy required for failure after the damage begins, and in brittle materials like ADIs, this energy was set to 0. Finally, since the residual fracture energy of ADIs was set to 0, when the strain of a single element reached the fracture strain of 0.064, the element was deleted, indicating that the fracture occurred at that element.

## 3. Results

### 3.1. Tensile Test Results

**Figure 4:**
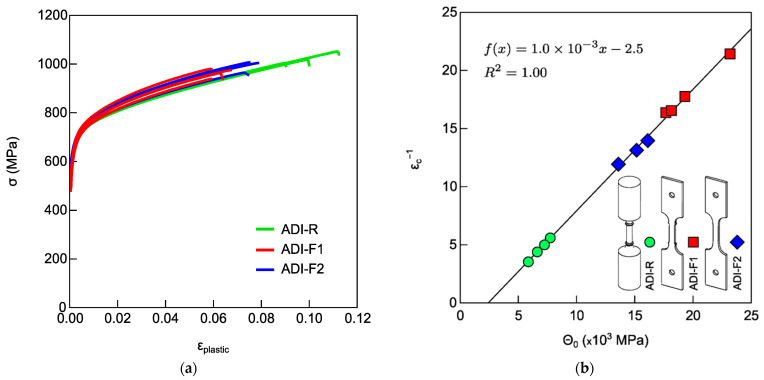
Tensile flow curves of ADI800 with chemical composition of ADI_1 (0.7%wt. of Ni): (**a**) round tensile specimens (ADI-R), flat tensile specimens with ridges (ADI-F1) and flat tensile specimens without ridges (ADI-F2); (**b**) MAD with Voce parameters from the fitting of the tensile flow curves of Figure 4a.

To assess the effect of specimen geometry on the ADI stability, tensile tests were carried out on tensile flat specimens with ridges (ADI-F1), without ridges (ADI-F2) and from round tensile specimens (ADI-R). The chemical composition of all the tested specimens was ADI_1 (0.7%wt. of Ni), so the specimen geometry was the only variable. The tensile flow curves are reported in Figure 4a. Figure 4b shows the MAD of the flow curves in Figure 4a. As seen in Figure 4a, the tensile flow curves were scattered in terms of both ductility and strain-hardening behavior. Figure 4b shows that the Voce data from all three tensile specimen geometries lie on the same best-fitting line. Moreover, the round tensile specimens exhibited the lowest Voce parameter values (average 1/ε_c_ = 4.6 ± 0.9), while the flat specimens with ridges had the highest values (average 1/ε_c_ = 18.0 ± 2.3). The intermediate values (average 1/ε_c_ = 13.0 ± 1.0) corresponded to flat specimens without ridges.

Besides the confirmation that flat geometry (ADI-F2) affected the tensile flow behavior of ADI800 as suggested in [43], it was found that the ridges in ADI-F1 further affected the strain-hardening behavior of ADI800, with a significant increase in Voce parameter values with respect to round specimens (ADI-R) and tensile flat specimens without ridges (ADI-F2). Also, the ductility was affected by both the specimen geometry and the presence of ridges in the flat tensile specimens, resulting in an average value of 6.2 ± 0.4% for the specimens with ridges (ADI-F1), 7.7 ± 0.3% for the specimens without ridges (ADI-F2), and 11.3 ± 1.0% for the round specimen (ADI-R). Fractures occurred close to the ridges for the tensile specimens ADI-R and ADI-F1, while fractures occurred in the gauge of the specimens ADI-F2.

**Figure 5:**
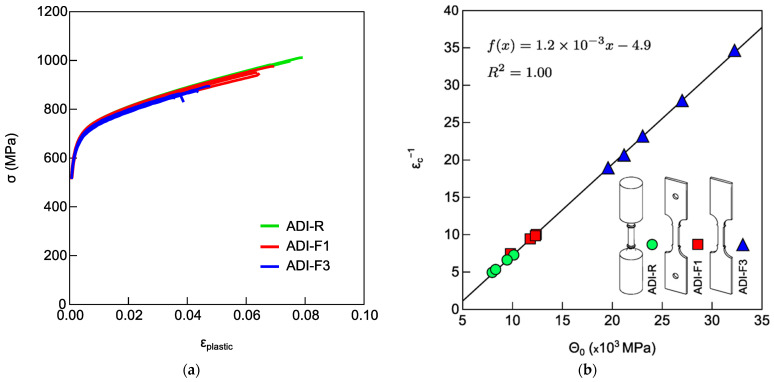
Tensile flow curves of ADI800 with chemical composition ADI_2 (1.5%wt. of Ni): (**a**) round tensile specimens (ADI-R), flat tensile specimens with ridges and with pin-hole (ADI-F1) and wedge grips (ADI-F3); (**b**) MAD with Voce parameters from the tensile flow curves of Figure 5a.

Tensile tests were carried out on flat specimens with a ridges and pin-hole gripping system (ADI-F1), with ridges and wedge gripping system (ADI-F3), and round tensile specimens (ADI-R). The chemical composition of all the tested specimens was ADI_2 (1.5%wt. of Ni), so the gripping system was the only variable. The tensile flow curves are presented in Figure 5a, while in Figure 5b, the MAD of the Voce parameters found from the tensile flow curves in Figure 5a is reported. In Figure 5a, the tensile flow curves appeared uniformly spread, differing in both ductility and strain-hardening behavior. In Figure 5b, the difference in strain-hardening behavior of the three kinds of specimens were evident, as the Voce parameters from the flat specimens with wedge grips (ADI-F3) were significantly higher than the other two tensile configurations (average 1/ε_c_ = 25.1 ± 6.3), and by far higher than the Voce parameters from the round tensile specimens (average 1/ε_c_ = 6.1 ± 1.1), while flat tensile specimens with ridges and pin-hole grips (ADI-F1) had intermediate Voce parameters values (average 1/ε_c_ = 9.2 ± 1.2). Moreover, the ductility was affected by both the specimen geometry and the gripping system, resulting in an average value of 4.0 ± 0.5% for the specimens with wedge grips (ADI-F3), 6.4 ± 0.4% for the specimens with pin-hole grips (ADI-F1) and 8.1 ± 0.7% for the round tensile specimens (ADI-R). Fractures occurred close to the ridges for all the tensile specimens, ADI-R, ADI-F1 and ADI-F3.

**Figure 6:**
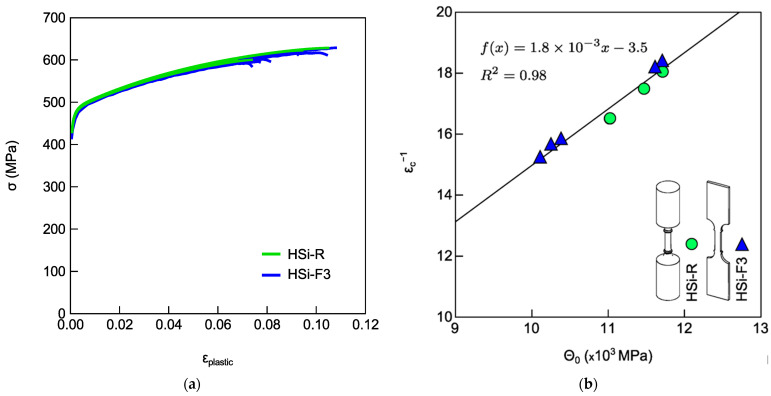
(**a**) Tensile flow curves from round tensile specimens (HSi-R) and flat tensile specimens with wedge grips (HSi-F3); (**b**) MAD with Voce parameters from the tensile flow curves of Figure 6a.

The experimental results from tensile tests in Figure 5 indicated that the largest difference in ductility and tensile strain-hardening behaviors in ADIs occurred between the round tensile specimens with threaded heads (ADI-R) and the flat tensile specimens with a wedge gripping system (ADI-F3). Further tensile tests were carried out on high-silicon strengthened ductile iron (HSi 4.2%wt. of Si, chemical composition in Table 1) to assess whether the tensile specimen geometry and gripping system alone could affect the tensile results, or if the observed difference in ductility and strain-hardening behavior is related to the ausferrite stability in ADI material. These tensile tests were performed on round specimens (HSi-R) and flat tensile specimens with ridges and a wedge gripping system (HSi-F3). In Figure 6a,b, the tensile flow curves and the MAD of the Voce parameters from the flow curves in Figure 6a are presented. As shown in Figure 6b, the data lie on the same line with a homogeneous distribution and similar Voce parameters (with average 1/ε_c_ = 17.2 ± 0.8 for HSi-R and average 1/ε_c_ = 16.3 ± 1.6 for HSi-F1). This finding indicated that the different specimen geometries did not affect the tensile plastic behavior of HSi material. Furthermore, the ductility of round tensile specimens (HSi-R) and flat tensile specimens with a wedge gripping system (HSi-F3) was similar, with an average value of 17.2 ± 0.8% for HSi-R and 16.3 ± 1.6% for HSi-F3 specimens. Fractures occurred in the gauge centers of all tensile specimens, HSi-R and HSi-F3.

### 3.2. DIC Results

**Figure 7:**
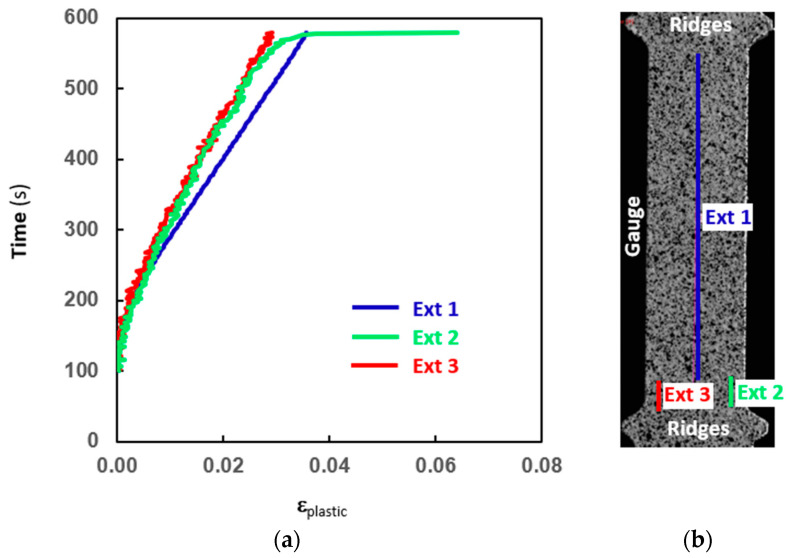
Time vs. true strain for the three different virtual extensometers (**a**) and virtual extensometers set for ADI-F3 sample tested with DIC system (**b**).

**Figure 8:**
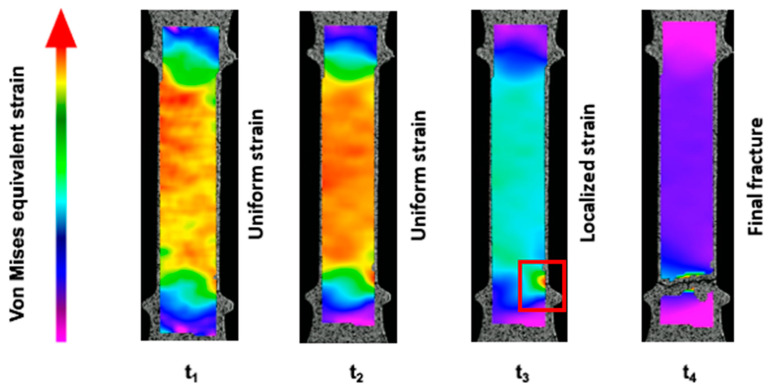
Evolution of strain measured by DIC during tensile testing of ADI-F3 samples. *t*_1_ = 248 s and *t*_2_ = 564 s: uniform plastic deformation in the gauge between the ridges; *t*_3_ = 572 s: the beginning of localized deformation below the lower ridge (red square) measured by Ext_2; *t*_4_ = 580 s: final fracture.

In Figure 7, an example of the distribution of virtual extensometers used with the DIC system in a flat tensile specimen of ADI-F3 (chemical composition ADI_2) and the corresponding time vs. elongation plots are reported. Through the virtual extensometers, the true strains between two selected measuring points are given, similarly to a regular extensometer, obtaining true stress–strain curves. These data are originally in total strain, but by identifying Young’s modulus, the data are separated into elastic and plastic components according to the procedure reported in Section 2.2. Ext_1 was extended through the whole tensile gauge length, and was used during the test for controlling the strain rate (nominally set to 10^−4^ 1/s). The extensometers Ext_2 and Ext_3 were selected after testing for localized strain measurements. Ext_2 concerned a gauge area close to the section where localized deformation occurred and later the final rupture started, while Ext_3 concerned the area where the very final rupture happened; see Figure 7b and Figure 8. In fact, in Figure 8, DIC images selected at t_3_ and t_4_ are reported to show where and when the fracture started (t_3_) and finally occurred (t_4_), which helps to visualize what is shown in Figure 7. In the images, the spatial distribution of von Mises true strains is reported over all the data points/virtual strain gauges between the points in the entire area of interest. The observations are always in total strain, and the virtual strain gauges measure the strains in all three directions. So, all three components of strains are then combined into von Mises equivalent strains. The colored arrow beside is reported as a qualitative scale of the von Mises equivalent strains along the flat tensile gauge.

Ext_1 vs. time in Figure 7a reported the total gauge strain behavior, achieving a final total true strain of 0.036 after 580 s. The local strains from the extensometers Ext_2 and Ext_3 were overlapping and quite lower than the strain from Ext_1 until the time of about 572 s, when localized deformation occurred close to the lower ridge of the specimen (Ext_2 in Figure 7b). The local deformation of Ext_2 kept increasing significantly up to a final local plastic true strain of 0.064 after 580 s. The true plastic strain vs. time where the final fracture formed was measured by Ext_3, and increased regularly and slightly during the localized deformation at Ext_2. The final fracture at Ext_3, indeed, formed suddenly at 580 s, and a final plastic strain of 0.029 was measured by Ext_3, by far lower than the localized deformation in Ext_2. So, in Figure 8, the deformation was quite uniformly distributed in the gauge from the beginning of the plastic regime, about t_1_ = 248 s, until about t_2_ = 564 s, while at about t_3_ = 572 s, clear localized deformation occurred and increased until the final fracture at t_4_ = 580 s. So, even if high localized plastic strain close to the ridge developed significantly (Ext_2) to trigger the final fracture, the plastic strain was confined there, and the final fracture occurred suddenly throughout the whole cross-sectional area without apparent plastic strain (Ext_3). This occurred in all five ADI-F3 specimens, and the final strain data are summarized in Table 3.

### 3.3. XRD Results

**Figure 9:**
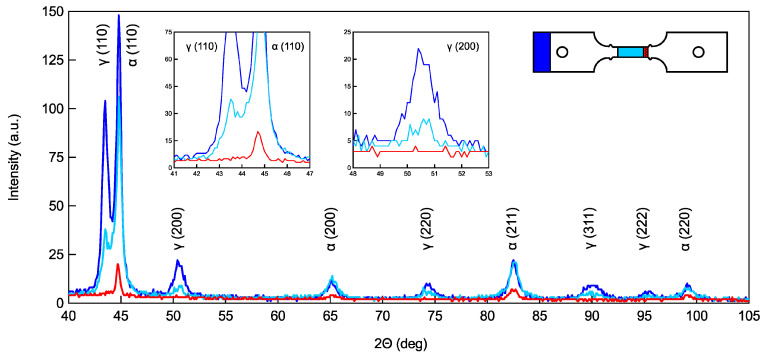
Typical XRD patterns obtained from three different positions in the flat specimens for a selected tensile test of ADI-F3 with chemical composition ADI_2, 1.5%wt. of Ni; blue line from the as-received non-deformed tensile specimen; light blue line from the deformed gauge length; red line from the fracture surface.

XRD measurements were carried out in different positions of selected flat tensile specimens, ADI-F1 and ADI-F3. The advantage of limiting the XRD measurements to the flat tensile geometry was that XRD was carried out only on the flat surface of the specimens without preparation, which indeed could introduce alteration of the austenite and martensite volume fractions. In fact, the round tensile specimens had to be cut, ground with sandpaper and polished before diffraction with the risk of producing martensite. As reported in Section 2.4, the estimated depth of XRD observation with Equation (2) is quite small, i.e., about 3.5 μm. So, surface conditions are critical for having reliable XRD measurements, and using only the flat tensile specimens, granted that the evolution of austenite to martensite with straining was reliable. Only the chemical composition ADI_2 was analyzed, as the volume fractions of metastable austenite depend significantly on the chemical composition of ADI. The flat tensile geometry ADI-F1 could give a correlation between the deformation in the gauge, while the ADI-F3 specimens that were tested with wedge grips and the DIC system could also give quantitative information on the local strain at the fracture. In [57,58], where investigations on austenite to martensite were carried out in TRIP austenitic steels, a significantly higher volume fraction of transformed martensite was reported on the fracture surface, which was higher than in the gauge of the tensile specimens. However, to the authors’ knowledge, a quantitative correlation between strain and austenite evolution, also considering the fracture surfaces, which was possible with the DIC technique, as reported in the present investigation, has never been described before. Typical XRD patterns from a flat tensile specimen of ADI-F3 are reported in Figure 9. The patterns showed that the ratio of the relative intensities of the γ_CH_ peaks compared to the α peaks in the as-received undeformed ausferrite (blue line) was significantly higher than that observed in the deformed gauge length (light blue line), where the average deformation was about 0.040. Interestingly, in the XRD pattern next to the fracture surface (red line), the γ peaks disappeared completely, and only α (and α’) peaks survived, suggesting that all metastable austenite fully transformed into martensite.

**Figure 10:**
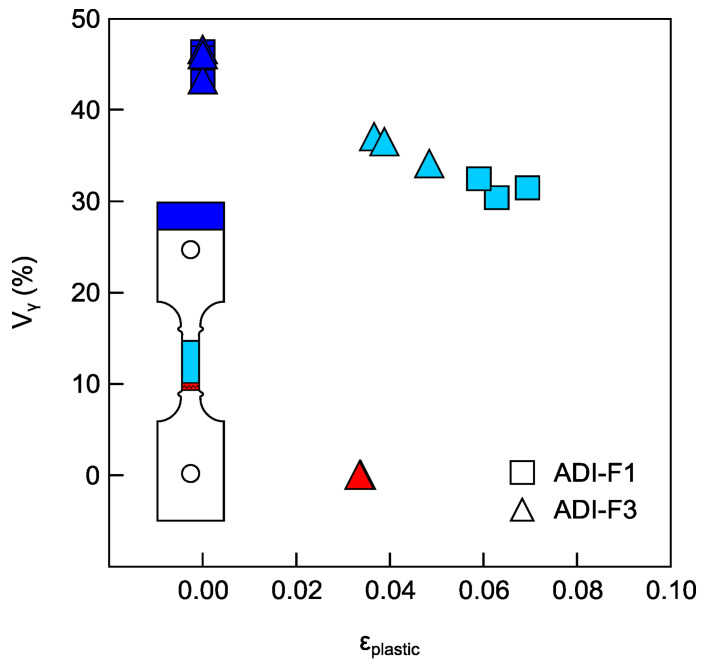
Volume fraction of metastable austenite versus plastic strain for the composition ADI_2: squared data points from flat tensile specimens with ridges and pin-hole gripping system (ADI-F1); triangle data points from flat tensile specimens with ridges and wedge gripping system (ADI-F3); blue line from the as-received non-deformed tensile specimen; light blue line from the deformed gauge length; red line from the fracture surface.

The volume fractions of metastable ausferrite (V_γ_) for ADI_2 composition, measured from different flat tensile specimens, were calculated according to the procedure reported in Section 2.4. and plotted as a function of the plastic strain (*ε_p_*). In Figure 10, the plot of V_γ_ vs. *ε_p_* is reported. The square data points correspond to the three flat tensile specimens with ridges and a pin-hole gripping system (ADI-F1), while the triangle data points represent the three flat tensile specimens with ridges and a wedge gripping system (ADI-F3). For the ADI-F1 tests, only strains derived from gauge elongation measurements were considered. On the other hand, for the ADI-F3 tests, DIC data were available, allowing the use of plastic strain values obtained from the entire gauge length (via virtual extensometer Ext_1 in Figure 7a), as well as the local strain at the fracture site via virtual extensometer Ext_3 in Figure 7a, and data reported in Table 3. With increasing plastic strain, the volume fraction of metastable austenite decreased significantly from an average of 45 ± 3% in the non-deformed conditions, dropping to below 40% at approximately 0.040 strain in the gauge length, and reaching zero at the fracture surface, where the final plastic strain was around 0.036. Therefore, the findings indicated that, during deformation, austenite transformed into martensite because of its mechanical instability under loading. In [57,58], where investigations on austenite to martensite were carried out in TRIP austenitic steels, a significantly higher volume fraction of transformed martensite was always reported on the fracture surface, which was higher than the volume fraction of martensite found in the gauge of the tensile specimens.

### 3.4. FEM Results

**Figure 11:**
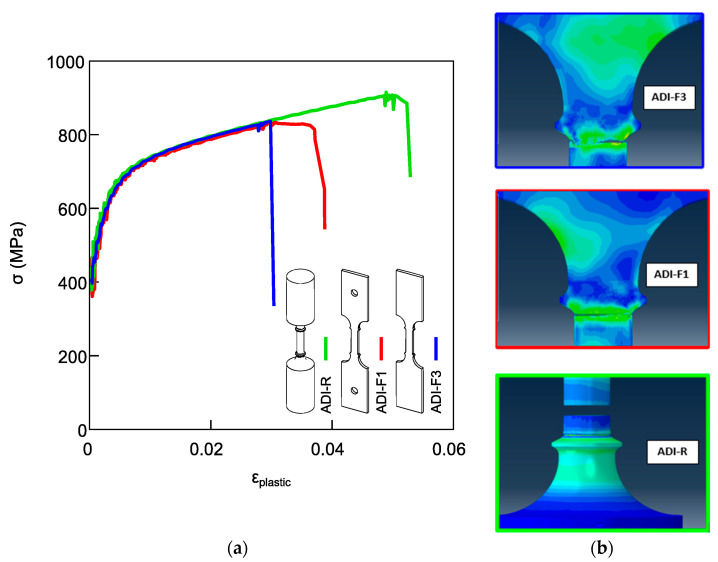
(**a**) FEM tensile flow curves of ADI800 with chemical composition ADI_2 (nominal 1.5%wt. of Ni) from round tensile specimen (ADI-R—green line), flat tensile specimen with pin-hole gripping system (ADI-F1—red line) and flat tensile specimen with wedge gripping system (ADI-F3—blue line); (**b**) simulations of the final rupture for the three different tensile specimen geometries, showing that the fractures occurred close to the ridges consistently to the experimental findings.

In Figure 11, the results from FEM simulations of ADI800 with a chemical composition of ADI_2 are reported. In Figure 11a, the tensile flow curves obtained from the three different geometries and gripping systems (ADI-R, ADIF1 and ADI-F3) matched the experimental results very well, describing the proper ductility trend reported for ADI_2: the highest ductility was observed in the round tensile specimen geometry (ADI-R); the flat tensile specimen with a pin-hole gripping system (ADI-F1) exhibited intermediate ductility, while the lowest elongation at fracture was related to the flat specimen with a wedge gripping system (ADI-F3). As shown in Figure 11b, the simulations predicted fracture locations near the ridges for all cases, in agreement with the experimental observations, thus supporting the accuracy of the simulations.

**Figure 12:**
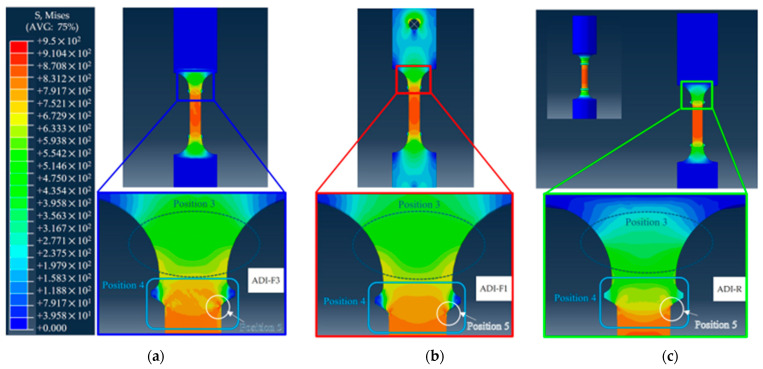
von Mises equivalent stress distributions of the three tensile specimens after a plastic strain of 0.034: (**a**) flat tensile specimen with ridges and wedge grips (ADI-F3); (**b**) flat tensile specimen with ridge and pin-hole grips (ADI-F1); and (**c**) round tensile specimen (ADI-R). Zoom sets at the ridges of the tensile specimens for the three different tensile geometries are reported.

In Figure 12, the von Mises equivalent stress distribution for the three tensile configurations AD-R, ADI-F1 and ADI-F3 is reported. The tensile specimens with threaded grip (ADI-R) and wedge grip (ADI-F3) did not show any stress at the specimen shoulders since they were not deformed. Particularly, the wedge grip in ADI-F3 did not allow the shoulders to deform elastically, introducing horizontal constraints that prevent the shoulders from any contraction. Figure 12’s zoom sets present the von Mises stress distributions near the ridges of the different tensile specimen geometries and gripping systems at the same plastic strain levels. At the same plastic strain of 0.034, the stress intensification was found to be the lowest for the round tensile (ADI-R) and the highest for the flat tensile specimen with wedge grips (ADI-F3). Specifically, at position 5, the von Mises equivalent stress values were 826.06 MPa, 871.72 MPa and 904.82 MPa for ADI-R, ADI-F1 and ADI-F3, respectively. These results indicated that not only the specimen geometry, but also the gripping system played a significant role in the degree of stress intensification near the ridges. The higher stress concentration observed in ADI-F3, associated with the wedge gripping system, is consistent with its lower ductility, as shown in Figure 11a.

**Figure 13:**
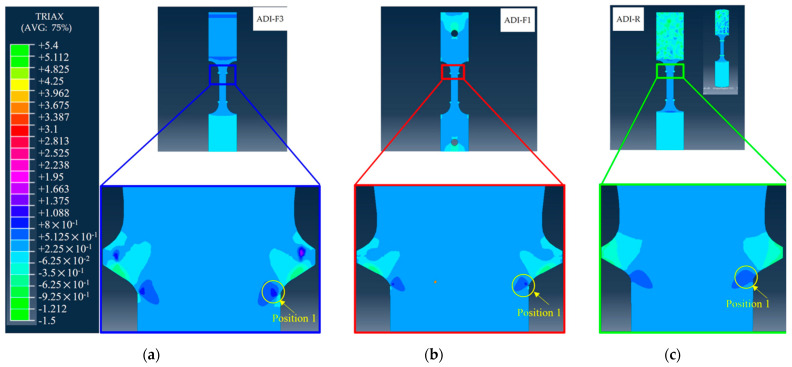
Triaxiality distribution at the ridges of the tensile specimens for the three different tensile geometries after a plastic strain of 0.034: (**a**) flat tensile specimen with ridges and wedge grips (ADI-F3); (**b**) flat tensile specimen with ridge and pin-hole grips (ADI-F1); (**c**) round tensile specimen (ADI-R).

Stress triaxiality is defined as the ratio of the hydrostatic stress to the von Mises equivalent stress. Different sample geometries and gripping systems can lead to differences in stress concentration, which in turn affect the magnitude of stress triaxiality. While the stress triaxiality for tensile-loading parallel gauges is 0.33, the stress triaxiality can be greater than 1 with complex geometrical features, where usually there are regions with strong stress concentrations (such as near the ridges of the tensile specimens and at the fracture tip), where the hydrostatic stress can be significantly higher than the von Mises equivalent stress. In Figure 13, the stress triaxiality results of FEM at the same strain of 0.034, like in Figure 12, are presented, resulting in triaxiality values at position 1 of 1.426 for the pulling configuration ADI-F3, 0.918 for ADI-F1 and 0.642 for ADI-R.

## 4. Discussion

### 4.1. Austenite Evolution During Tensile Testing

It has been reported that residual stresses [59,60,61] can pre-stress the material and affect the austenite stability during subsequent thermal or mechanical loading. The magnitude of the final applied stress is a complex interplay between the residual stresses from the production processing and the tensile load during the austenite-to-martensite transformation, enhancing or hindering the TRIP effect. Also, the crystallographic orientation of austenite grains significantly affects their stability against transformation, as reported in tensile deformation investigations. So, specific crystallographic texture components can promote the austenite-to-martensite transformation under tensile loading, while others can hinder the transformation [59,62,63], increasing the stability of austenite. In the present investigation, however, no specific crystallographic texture was found, indicating that a proper inoculation was carried out during the melt treatment in the foundry, producing fine equiaxed grains. Therefore, after austempering at 380 °C for one hour [43], the ADIs were also then cooled slowly according to the common austempering production route to avoid any residual stress building up, which could affect the ausferrite stability. Furthermore, the flat tensile specimens were obtained by slicing the ADI 25 mm Y-blocks along the same block direction, and the surfaces were finished, reducing as much as possible any residual stress at the specimens’ surface. Therefore, it could be assumed that the flat tensile specimens featured the same amount of retained austenite and distribution of residual stresses. As such, these parameters could not play a role in affecting the comparison of mechanical properties from one tensile specimen to another.

The XRD measurements obtained at different positions of the flat tensile specimens (Figure 9) and the calculations of the volume fractions Vγ of metastable ausferrite for the ADI_2 (1.5%wt. of Ni) reported in Figure 10 confirmed that the austenite-to-martensite transformation occurred during tensile testing. Vγ decreased from an average of 45 ± 3% in the non-deformed conditions to about 35% after about 0.040 of plastic true strain, and further to about 30% after 0.070 of plastic true strain. On the fracture surface that corresponded to about 0.030 true strain according to the local DIC measurements reported in Table 3, the austenite disappeared completely. This last finding was consistent with results already reported in the literature [57,58] for investigations on austenite-to-martensite evolution in TRIP steels, where the volume fractions of austenite were always significantly lower on the fracture surface than in the tensile specimen bulk. However, a quantitative relationship between surface plastic true strains and austenite volume fractions based on the DIC technique has never been reported before, to the authors’ knowledge. The mechanical instability of austenite in ADIs has been reported to be highly dependent on the austenitization and austempering conditions. In [37,38,51,52], it was shown that the martensite was induced by strain, while in [27,53,54], no such strain-induced martensite formation was found in ADIs. Indeed, in [27], the authors reported that under constant austenitization conditions, ADI showed greater thermal and mechanical stability if austempered at lower austempering temperatures, with no martensite formation during tensile loading. However, as the austempering temperature was increased, the austenite became significantly less stable, and martensite appeared after cryogenic treatment or because of tensile loading. In the present investigation, the two investigated ADI800s were both heat-treated with an austenitization temperature of 875 °C for 2 h, and an austempering temperature of 380 °C for 1 h, which is consistent with the high temperature austempering and low stability of ausferrite reported in [27]. Investigations on austenite-to-martensite evolution during straining were carried out with quantitative evaluation through XRD [55] and neutron diffraction [56]: the strain was given through compression, as higher deformations could be achieved before fracture with this loading mode. In [55,56], a linear relationship was found between martensite volume fractions and compressive strain, up to about 40% of reduction. In the present work, the decrease in austenite volume fraction did have a quite linear relationship with the plastic strain from the non-deformed conditions (Vγ = 45 ± 3%) up to 0.070 of plastic true strain (Vγ ≈ 30%), confirming the strain-induced austenite-to-martensite transformation.

However, at the fracture surface, the volume fraction of austenite was zero after an average plastic true strain of 0.30, lower than in the gauge, and, above all, no detectable strain development during the fracture was measured by the DIC technique. In fact, even if the extensometer Ext_2 measured a high localized plastic true strain close to the ridge, the plastic true strain at the very fracture measured by Ext_3 was by far lower than what experienced in the gauge (Ext_1) and, above all, Ext_3 did not report any increment during the sudden fracture event; see Figure 7 and Figure 8, and Table 3. This was an indication that the austenite-to-martensite evolution at the fracture was stress-induced, as no plastic true strain was measured. This finding is consistent with what is reported in [27], where the austenite stability of ADIs has been investigated in fracture toughness tests, reporting the reduction in austenite on the fracture surfaces of the CT specimens, where no significant plastic strain was found because of the plane strain conditions. Furthermore, the authors proposed that the stress concentrations and the high stress triaxiality ahead of a propagating fracture could lead to the stress-induced formation of martensite in ferrous alloys containing austenite. The finding of the present investigation in Figure 10 agrees with the model proposed in [27], and gives further and quantitative support to it, thanks to the DIC measurements reported in Figure 7 and Figure 8, and Table 3. So, as the fracture was propagating, the high stress and triaxiality at the moving tip of the crack caused the austenite to transform into martensite ahead of the ongoing fracture, resulting in a stress-induced austenite-to-martensite transformation.

### 4.2. Tensile Behavior and Ausferrite Instability

ADI800 was tested with different tensile specimens’ geometries and gripping systems for a total of at least four possible tensile testing configurations. Round and flat tensile specimens were machined, complying with different routes, which might have produced different local heating, differences in surface conditions, or the presence of different residual stresses. Indeed, if present, all of these did not affect the tensile behavior of the specimens made of high silicon DI, where the microstructure was fully ferritic and there was no phase transformation. In fact, the round tensile specimens (HSi-R) and flat tensile specimens with ridges and wedge grips (HSi-F3) had matching ductility properties and Voce parameters values. The tensile results reported in Figure 6 showed that the tensile curves of HSi-R and HSi-F3 overlapped with similar average ductility (9.74 ± 2.12% for HSi-R specimens and 10.50 ± 1.37% for HSi-F3), and the Voce parameters from round and flat tensile geometries were randomly distributed on the same best fitting line in MAD, as shown in Figure 6b, resulting in matching Voce parameters (average 1/ε_c_ = 17.2 ± 0.8 for HSi-R and 1/ε_c_ = 16.3 ± 1.6 for HSi-F3). Therefore, it can be concluded that the different pulling geometries and production routes of the specimens did not affect the tensile behavior, per se, and the findings concerning ADI800 were truly related to austenite-to-martensite transformation, that is, the austenite instability because of mechanical loading. From the results reported in Figure 4 and Figure 5 on ADI800, it was found that the flat tensile specimens’ geometry decreased the ductility and affected the strain-hardening response, thus increasing the Voce parameters values in MADs (see Figure 4b and Figure 5b). This strain-hardening behavior has been associated with ausferrite instability in several publications [12,13,43]. The largest difference in ductility and strain-hardening behavior was found for the ADI-R, which had the best ductility (for instance, 8.1 ± 0.7% in ADI_2) and lowest Voce parameters values (1/ε_c_ = 6.1 ± 1.1 in ADI_2) in the MAD, while the flat tensile specimens with ridges and wedge grips ADI-F3 had the lowest ductility (4.0 ± 0.5% in ADI_2) and the highest strain-hardening Voce parameters (25.1 ± 6.3 in ADI_2). ADI-F1 had an intermediate ductility (6.4 ± 0.4% in ADI_2) with Voce parameters (9.2 ± 1.2 in ADI_2) between ADI-R and ADI-F3.

For the rationalization of the experimental tensile behaviors, FEM simulations were needed, as the gripping system was also a constitutive part of the pulling configuration and significantly affected the ductility and the strain-hardening behavior that indicated the ausferrite instability. The FEM simulations were carried out by using a single experimental flow curve and von Mises model from a round tensile specimen and composition of ADI_2 (1.5%wt. of Ni). So, no indication about the different strain-hardening behavior could be gathered, but the simulations were paramount for having indications on the contribution of the different gripping systems to the stress and triaxiality distribution along the tensile specimen gauge, and particularly near the ridges, to understand the influence of the tensile specimen geometry and gripping system on the ductility and fracture behavior of the three different tensile pulling configurations. This could simplify the rationalization of the different strain-hardening behaviors that are related to the ausferrite instability because of the austenite-to-martensite transformation found through XRD analysis in Figure 10. In Figure 12, the simulation images of the three tensile configurations show that the stress intensification at the ridges was different for the three pulling configurations with ridges: for instance, at the same strain of 0.034, just before the fracture of the more brittle pulling configuration (ADI-F3), the stress was the lowest for the ADI-R specimen (826.06 MPa), intermediate for the ADI-F1 (871.72 MPa) and the highest for the ADI-F3 (904.82 MPa), which explains the experimental fracture at the ridges and the different ductility for the three tensile geometries. Furthermore, in Figure 13, the stress triaxiality coefficient in the same position near the ridges was 1.426 for the pulling configuration ADI-F3 with wedge grips, 0.918 for ADI-F1 and 0.642 for ADI-R. This high stress triaxiality state exacerbated the brittle fracture tendency of the material in ADI-F1 and ADI-F3. The FEM simulation results are consistent with the ductility trend and the locations of the final fractures reported in Figure 11.

**Figure 14:**
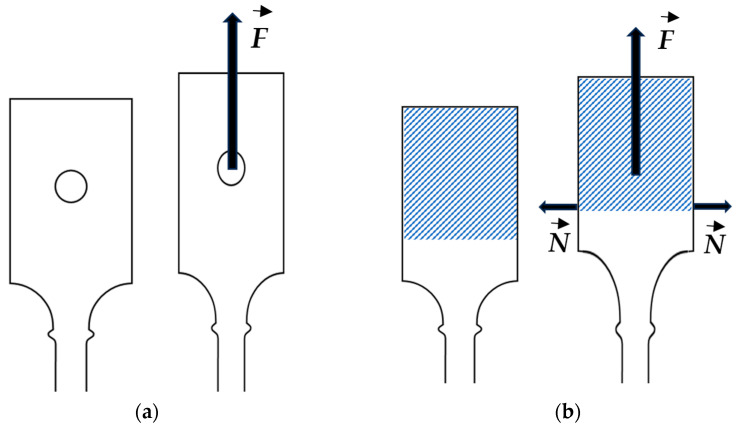
Rationalization of the loading conditions in the tensile tests: (**a**) pin-hole gripping system; (**b**) wedge gripping system with the appearance of constraints $\vec{N}$.

Indeed, the different behavior of flat tensile specimens with pin-hole and wedge gripping systems can be rationalized with the help of the sketch in Figure 14. During tensile loading, a pulling force $\vec{F}$ is imposed to the flat tensile specimen: for the pin-hole gripping system (Figure 14a), the flat tensile specimen heads are deformed elastically and the horizontal part of the specimen head can contract; conversely, for the wedge gripping system (Figure 14b), the wedges with gripping stop the elastic strain of the specimen heads, producing horizontal constraints $\vec{N}$ due to the mismatch of deformation with the free material volume. The wedge gripping systems inhibited the lateral elastic contraction of the specimen heads and partially constrained the contraction in the regions between the heads and the ridges, leading to higher stress triaxiality at the ridges, and increasing the stress intensifications with respect to the pin-hole configuration reported in Figure 14a.

In the present investigation, there is a correlation between ductility trend and strain-hardening behaviors in the investigated ADI800s. For the lowest ductility, the highest Voce parameters were found for the flat pulling configuration ADI-F3 with ridges and wedge grips, and, vice versa, for the highest ductility, the lowest Voce parameters were measured for the round pulling configuration ADI-R with ridges. According to [12,13,40,41,42], the increase in strain-hardening Voce parameters is related to austenite-to-martensite transformation that was indeed found in the present investigation. In addition, in Section 4.1, it was proved that high stress intensification and triaxiality could trigger the austenite-to-martensite transformation at the fracture. So, consistently in ADI-F3, the high stress intensifications and triaxiality at the ridges could explain the higher instability of ausferrite than the one observed for the round specimens ADI-R, resulting in a dramatic increase in the Voce parameters of ADI-F3 in MAD; see Figure 5b. The strain-hardening behavior of ADI-F1, which had stress intensification and triaxiality between ADI-R and ADI-F3, had Voce parameters in between, indicating an intermediate ausferrite instability. In support of this explanation, the flat tensile specimens with a pin-hole gripping system but without ridges (ADI-F2) had a ductility between ADI-R and ADI-F1 with a strain-hardening behavior in between; see Figure 4b. The elimination of the ridges that are stress and triaxiality raisers caused an improvement of ausferrite stability compared to ADI-F1.

So, the experimental results supported by the FEM simulations show that the tensile specimen geometry is not the only parameter to be considered when pulling metallic materials, but the whole experimental tensile configuration with the gripping system has to be analyzed, as it can affect the tensile properties. If in ductile materials without phase transformation, like HSi DIs, this effect had not been detected, while for a brittle material like the investigated ADI800, where the microstructure is unstable and austenite-to-martensite transformation can occur because of tensile loading, the effect is dramatic and must be considered. To the authors’ knowledge, these findings on the ausferrite stability with mechanical loading have never been reported in the literature before.

### 4.3. Ausferrite Instability and Stress Raisers

For the tensile specimens’ geometries and gripping systems, the simulation results have captured that only due to differences in tensile specimen geometry (such as round vs. flat) and gripping constraints (such as pin-hole vs. wedge gripping), the stress distribution of the specimens in Figure 12 and triaxiality in Figure 13 and the fracture location (near the ridges) exhibit significant differences, which are consistent with the observed ductility and austenite instability sequence in the experiments. So, from the results based on Voce analysis of strain-hardening analysis in MADs, XRD measurements and FEM simulations, it was found that the stress raisers, such as the ridges of the tensile specimens and the gripping systems, significantly affected the ductility and the strain-hardening behavior of ADI800, which was related to the instability of ausferrite. In fact, with HSi specimens with round geometry (HSi-R) and flat geometry with ridges and wedge grips (HSi-F3), there was no difference in ductility and strain-hardening behavior (see Figure 6). The absence of austenite on the fracture surface, as revealed by the XRD measurements in Figure 9 and Figure 10, provided further evidence that the stress intensification and triaxiality drove the austenite-to-martensite transformation. Strain-induced martensite in the plastically deformed gauge section, and stress-induced martensite at the fracture surface can explain these findings.

It is interesting to note that the flat geometry affected the instability of ausferrite, as reported in Figure 4b for the flat tensile specimens ADI-F2 without ridges with pin-hole grips, having average Voce parameter values (1/ε_c_ = 13.0 ± 1.0) significantly higher than the round tensile specimens (average 1/ε_c_ = 4.6 ± 0.9). However, if compared to the effects of ridges and wedge grips, this was a second-order effect, even though detectable. It is known that surface defects and residual stresses produced during austempering, or subsequent processing, can induce ausferrite instability with austenite-to-martensite transformations [64,65,66]. Indeed, consistent with the findings reported in the present investigation where the stress intensification and triaxiality at the ridges with different gripping systems resulted relevant in affecting the strain hardening of ADI800 and so the ausferrite stability, the presence of any stress raiser at the surface of specimens or structural feature that may increase the stress concentration, could induce the nucleation of a crack or ausferrite instability at that specimen region. Therefore, surface conditions of the tensile specimens were relevant, and the flat geometry offered a higher surface to bulk volume ratio than in a solid tensile specimen, with a higher probability to trigger austenite-to-martensite transformation: this transformation could expand from the surface to the gauge interior.

For instance, the graphitic nodules can also be the sites of stress concentration. It was shown that in DIs, the graphite nodules and micro-shrinkage cavities could lead to a reduction in tensile strength [67,68,69]. Moreover, fatigue crack initiation is generally observed at the graphite nodules and porosities. Their size, shape and distribution, such as nodule inter-distance in nodule clusters and their position relative to the surface of the tensile specimen, are important, since these defects are more effective when they are at the surface. However, in ADIs [69], micro-segregations occur close to the interface between graphitic nodules and metallic matrix, with an increase in manganese and molybdenum content that can, in turn, produce blocky unstable austenite. Therefore, graphitic nodules represent potential sites for austenite-to-martensite transformation due to both their roles as stress raisers and chemical micro-segregations. It is well known that the voids of graphitic nodules at the surface of a tensile specimen are more effective stress raisers. So, the higher instability of ausferrite with the flat tensile specimen could be rationalized by considering that the number of voids of graphitic nodules per unit area at the external surface of the gauge area is higher in a flat tensile specimen than in a round tensile specimen. For instance, considering a round tensile specimen geometry (gauge diameter *d*_o_ = 5.6 mm and length *l*_o_ = 28.0 mm) with a nodularity of 100 nodules/mm^2^, the number of nodules at the gauge surface per unit area is 71.4 nodules/mm^2^. On the other hand, for a flat tensile specimen used in the present investigation (gauge thickness *t*_o_ = 1.5 mm, width *w*_o_ = 5.0 mm and length *l*_o_ = 20.0 mm), the number of nodules at the gauge surface per unit area is 173.3 nodules/mm^2^ for the same nodule count of 100 nodules/mm^2^. Of course, this rationalization can be extended to any possible defect or microstructure heterogeneity that could be present at the surface of the tensile specimens. In the case of the HSi ductile iron, no austenite-to-martensite transformation was observed, and the material exhibited good ductility, as localized deformation with necking was always achieved. Consequently, the specimen geometry HIS-F3 (ridge and/or wedged grips) did not affect the ductility and the strain hardening of HSi. This can be confirmed by the overlapping of the tensile flow curves of flat and round specimens in Figure 6a, as well as the random distribution of Voce parameters in the MAD (Figure 6b).

Finally, it is worth mentioning that the size effect could play a role in mechanical performance, which is an important concern for practical applications. The size effect consists in the tensile specimen size dependence of material properties, and is related to the material’s microstructure and, eventually, the inhomogeneous distribution of its phases. In TRIP steels, for instance, the volume fraction, morphology, crystallographic orientation, and stability of austenite are relevant for the austenite-to-martensite transformation [62,63]. When specimen size is reduced significantly, the austenite with all its microstructural features mentioned above may no longer be similarly represented in the different specimens’ geometry or size, resulting in different material responses. In fact, the tensile size effect on material plastic behavior is usually rationalized by surface-layer models, where the surface properties are assumed to be different from the bulk ones. Considering materials strength, for instance, the surface grains are assumed to be softer than the bulk grains [70,71], when the ratio of the surface to the bulk increases with decreasing tensile specimen size, then the specimen becomes weaker. It is noteworthy that in the qualitative rationalization proposed by the authors on the present different austenite instability in round and flat tensile specimens, a surface-layer-model approach is indeed implemented, suggesting that the graphitic nodules (or surface scratch or any other surface stress raiser) may be austenite instability trigger sites, and their number is significantly higher in the flat tensile geometry than in the round one for the same microstructure. However, the size effect was out of the scope of the present study, and the current results suggest that the tensile size effect on ausferrite instability should be investigated further in the future.

## 5. Conclusions

The effects of tensile specimen geometry and gripping system on the stability of austenite in ADI800 were investigated by combining tensile testing, DIC technique for precise local strain measurements and the evolution of the austenite-to-martensite transformation with strain through XRD technique. FEM simulations were carried out to support the rationalization of the experimental findings, and through the same constitutive model, the input captured the differences in the investigated tensile specimens’ geometries and gripping systems.

1. In the deformed gauge of the flat tensile specimens, the austenite-to-martensite transformation was strain-induced, while at the fracture surface, the austenite disappeared completely with no recorded strain development, so that the austenite-to-martensite transformation at the fracture was explained as stress-induced because of the stress intensification and triaxiality at the advancing cracks.
2. FEM simulations found that the ridges limiting the tensile gauge and the wedge gripping system produced significant stress intensification and triaxiality, causing a substantial reduction in ductility, capturing the trend of ductility and the fracture locations for all the investigated pulling configurations.
3. The austenite instability, consistent with the rationalization of the triaxiality effect on the austenite-to-martensite transformation, was also supported by FEM simulations and rationalized as caused by high stress concentrations and triaxiality due to the presence of the ridges and wedge gripping systems, because the wedges produced some constraints to the elastic lateral contractions of the flat tensile specimen heads.
4. The flat tensile specimen geometry affected the ductility, though it was less significant than the pulling configurations with ridges. Consistent with the fact that the structural specimens’ features (ridges and grips) caused the reduction in ductility and increased the ausferrite instability, this finding was explained as being caused by the surface imperfections that could act as stress raisers, and graphitic nodules at the surface of the specimens could be one of these.

In conclusion, when the tensile properties of ausferrite and ADIs are investigated, it is essential to assess the effects of tensile specimen geometry and of the gripping system, as both have been proven to significantly influence the austenite-to-martensite transformation, i.e., the instability of ausferrite that is related to the plastic behavior of ADIs.

## Figures and Tables

**Table 1:** Chemical compositions of the investigated ductile irons (%wt.).

Composition	C	Si	Mn	S	P	Mg	Cu	Ni	Fe	C_Eq_
ADI_1	3.52	2.62	0.31	0.013	0.036	0.050	0.74	0.74	Bal.	4.32 ^1^
ADI_2	3.52	2.54	0.36	0.012	0.042	0.037	0.70	1.53	Bal.	4.30 ^1^
HSi	3.54	4.20	0.13	0.006	0.014	0.045	-	-	Bal.	4.72 ^2^

^1^ C_Eq_ = C %wt. + 0.33 × Si %wt. + 0.30 × P %wt. [43]. ^2^ C_Eq_ = C %wt. + 0.28 × Si %wt. + 0.30 × P %wt. [46].

**Table 2:** Tensile specimen codes with the description of the relevant chemical compositions (ADI and HSi), tensile specimen geometry and gripping system.

Tensile Specimen Code	Description
ADI-R	ADI—Round geometry
ADI-F1	ADI—Flat geometry with ridges and pin-hole grips
ADI-F2	ADI—Flat geometry without ridges and pin-hole grips
ADI-F3	ADI—Flat geometry with ridges and wedge grips
HSi-R	HSi 4.2%wt. of Si—Round geometry
HSi-F3	HSi 4.2%wt. of Si—Flat geometry with ridges and wedge grips

**Table 3:** DIC strain information concerning the five ADI-F3 tensile specimens with chemical composition ADI_2 (1.5%wt. of Ni).

Tensile Specimen Number	Total Plastic True Strain at the Gauge (Ext_1)	Localized Plastic True Strain (Ext_2)	Plastic True Strain at Fracture (Ext_3)
1	0.037	0.074	0.034
2	0.039	0.081	0.033
3	0.048	0.067	0.034
4	0.044	0.076	0.028
5	0.036	0.064	0.029

## Data Availability

The original contributions presented in this study are included in the article. Further inquiries can be directed to the corresponding author.
